# Physiological variations of blood pressure according to gender and age among healthy young black Africans aged between 18 and 30 years in Côte d’Ivoire, West Africa

**DOI:** 10.14814/phy2.14579

**Published:** 2020-09-28

**Authors:** Edwige Siransy‐Balayssac, Soualiho Ouattara, Téniloh Augustin Yéo, Aya Liliane Kondo, Massiré Touré, Cyrille Serges Dah, Pascal Bogui

**Affiliations:** ^1^ Laboratoire de Physiologie et d'Explorations Fonctionnelles Unité de Formation et de Recherche en Sciences Médicales Université Félix Houphouët Boigny 01 BPV 34 Abidjan 01 COTE D'IVOIRE; ^2^ Service des Explorations Fonctionnelles Centre hospitalier universitaire de Yopougon 21 BP 632 Abidjan 21 COTE D'IVOIRE; ^3^ Service des Explorations Fonctionnelles Centre hospitalier universitaire de Cocody BPV 13 Abidjan COTE D'IVOIRE

**Keywords:** age, black African, blood pressure

## Abstract

In Africa, abnormal high blood pressure is common and affects young subjects. The risk of organ damage and mortality increases with blood pressure level. Therefore, the purpose of this study was to assess the blood pressure profile of a black African population aged between 18 and 30 years in Côte d’Ivoire. Five hundred fifty‐one healthy black African students, men and women, with sedentary lifestyle, aged between 18 and 30 years were selected. Systolic (SBP) and diastolic (DBP) blood pressures and heart rate were recorded after 5 min of rest. Regression models were used to estimate the effects of age, gender, and body mass index (BMI) on SBP and DBP. Each increase of 1 year in age and 1 kg/m^2^ of BMI is significantly associated, respectively, with an increase of 20% (*p* = .002) and 17% (*p* = .008) in the risk of having an SBP ≥ 130 mmHg. The same risk is 3.8 times greater for men than women (*p* = .01). Among subjects with SBP < 120 mmHg, men have an SBP 5.22 mmHg higher than women (*p* < .001). The increase in the age of 1 year is significantly correlated with a rise of 36% of having a DBP ≥ 85 mmHg (*p* = .0001). Also, in men population, the age increase of 1 year is associated with a rise of 41% of having a DBP ≥ 85 mmHg (*p* = .0001). Among young black African students aged between 18 and 30 years in Côte d’Ivoire, SBP is positively associated with male gender, age, and BMI. For DBP, it is only an increase with age.

## Abstract 2

Among young black African students, the increase in age is significantly associated with higher systolic and diastolic blood pressures. The risk of having higher systolic blood pressure is greater for men than women.

## 1 INTRODUCTION

Abnormal high blood pressure has been a public health problem for many years. In 2015, it affected 1.13 billion people worldwide (NCD, 2017). According to the World Health Organization, cardiovascular diseases are responsible for approximately 17 million deaths per year worldwide, of which 9.4 million (55%) are due to complications of abnormal high blood pressure (Lim et al., 2012; OMS, 2008). By 2025, researchers estimate that 29.2% of the adult population, or 1.56 billion people, will have abnormal high blood pressure, representing a 60% increase between 2000 and 2025 (Kearney, 2005).

In Africa, this abnormal high blood pressure has specific features. It is the leading cause of cardiovascular diseases, ahead of smoking, diabetes, and dyslipidemia (Steyn, 2005). It is more common, affecting 16 to 46 or even 60% of adults of 25 years old or above (Ataklte, 2015; Campia, Cardillo, & Panza, 2004; Dzudie et al., 2017; Gómez‐Olivé et al., 2017; Houehanou, Amidou, Preux, Houinato, & Lacroix, 2018; NCD, 2017). It affects young subjects, in majority between 31 and 55 years old (Ataklte et al., 2015; Campia et al., 2004; Desormais et al., 2019; Hendriks et al., 2012). It is characterized by higher blood pressure levels, early complications, and higher mortality estimated at 150 per 100,000 compared to 125 per 100,000 worldwide (Houehanou et al., 2018; Neaton, Kuller, Wentworth, & Borhani, 1984; Stamler, Stamler, & Pullman, 1967). By 2025, more than 150 million people in Africa will have an abnormal high blood pressure compared to 80 million in 2000 (Yaya & Kengne, 2014).

The prevalence of abnormal high blood pressure increases with age (Desormais et al., 2019; Hendriks et al., 2012; NCHS, 1966; US depth of health, 1977) and the risk of complications and mortality rises steadily with blood pressure level (Build, 1959; Report, 1977).

Faced with this pandemic, it is important to identify the blood pressure profile of our African populations by age group, in order to develop appropriate preventive strategies.

Therefore, the purpose of this study was to determine the blood pressure profile according to age and anthropometric parameters in a healthy black African population aged between 18 and 30 years, living in Côte d’Ivoire, West Africa.

## 2 MATERIALS AND METHODS

### 2.1 Ethical approval

This study was approved by the Ethics Committee of the Academic Hospitals of Yopougon (Abidjan, Ivory Coast) and complied with the guidelines set by the Declaration of Helsinki. All patients were informed about the purpose and procedures of the study and gave their written consent.

### 2.2 Population

The study population consisted of student volunteers from the Félix Houphouët‐Boigny University in Abidjan, Côte d'Ivoire. They were selected from October 2004 to August 2019. Among 728 volunteers, 551 (391 men and 160 women) were selected using inclusion and exclusion criteria through a questionnaire. They were all black Africans, aged between 18 and 30 years old and had a sedentary lifestyle. They did not take any medication, alcohol, coffee nor tea. We excluded subjects with known cardiovascular risk factors (hypertension, obesity, smoking, diabetes, and dislipidemia) and with signs, symptoms or history of cardiovascular, respiratory, and hematological diseases, which define them as healthy subjects in this study. Files with missing information were not retained. Women did not have any episodes of amenorrhea for at least 3 months. They were included regardless of their menstrual cycle phases. A previous study conducted in this black African women population found no significant difference in systolic blood pressure between phases of the menstrual cycle. Moreover, regardless of their menstrual cycle phases, all women had diastolic blood pressure below 80 mmHg (Balayssac‐Siransy et al., 2014).

### 2.3 Protocol study

In the trial study protocol, subjects are received in the morning, with an empty stomach, between 7 and 8 a.m. according to a pre‐established program, at the physiology, and functional explorations laboratory of the Medical Sciences unit of research and training in Abidjan (Côte d’Ivoire). On the test day, major stress, medical signs, and possible medication intake were searched. Body weight was assessed to the nearest 0.5 kg using an electronic scale. The patients were lightly dressed, with no belt on, no shoes, and empty pockets when body weight was measured. Height was recorded to the nearest 0.5 cm for each patient, using a vertical scale. Measurements of systolic (SBP) and diastolic (DBP) blood pressures and heart rate were taken using an electronic blood pressure monitor (OMRON M6 brand, Japan) with a cuff fixed on the non‐dominant arm. These parameters were recorded after 5 min of rest, in a seated position with back and arms supported, in a quiet and semi‐illuminated air‐conditioned room at 22°C.

### 2.4 Statistical analysis

The overall women and men populations were divided into four (4) groups according to the level of systolic and diastolic blood pressures based on the European Society of Cardiology (ESC) classification of blood pressure (Williams et al., 2018) (Tables 2 and 3). Because of the fewer sample size observed in some groups, SBP and DBP were each reclassified in two groups: normal group that included respectively subjects with SBP < 130 mmHg and DBP < 85 mmHg and abnormal group, respectively, with those with SBP ≥ 130 mmHg and DBP ≥ 85 mmHg.

Data were exported, cleaned, coded, and analyzed using R version 4.0.2. Descriptive analysis was conducted on both continuous and categorical variables. For continuous variables such as age, body mass index, heart rate, systolic, and diastolic blood pressures, data were summarized as means ± standard deviation. For categorical variables such as gender, SBP groups, and DBP groups, data were summarized using proportion and frequency. Wilcoxon–Mann–Whitney test and Kruskal–Wallis test were used to perform group comparison. Linear and logistic regressions were used to estimate the effects of age and anthropometric parameters on systolic and diastolic blood pressures changes and to evaluate their effects on the probability to fall in a specific systolic or diastolic blood pressure group. Analysis was performed at type I error level of 0.05.

## 3 RESULTS

### 3.1 Population characteristics

Characteristics of the study population are recorded in Table 1. Men are older with significantly higher systolic blood pressure (SBP) than women. Women have a significantly higher heart rate (HR) than men. Diastolic blood pressure (DBP) and body mass index (BMI) are similar in both genders (Table 1).

**TABLE 1:** Anthropometric and hemodynamic characteristics in the population‐based study

Parameters	Overall *(n = 551)*	Women *(n = 160)*	Men *(n = 391)*	*p* *(Women‐men)*
Age (years)	22.3 ± 2.5	21.2 ± 2.1	22.7 ± 2.5	<.0001
BMI (kg/m^2^)	21.4 ± 2.6	21.9 ± 3.6	21.2 ± 2	.42
SBP (mmHg)	111 ± 12	105 ± 10	114 ± 12	<.0001
DBP (mmHg)	70 ± 8	70 ± 7	70 ± 9	.88
HR (bpm)	66 ± 11	72 ± 12	63 ± 10	<.0001

Note

Data expressed as mean ± standard deviation

Abbreviations: <, less than; BMI, body mass index; DBP, diastolic blood pressure; HR, heart rate; SBP, systolic blood pressure.

### 3.2 Systolic blood pressure (SBP)

In Table 2, SBP is presented in four (4) groups, optimal (<120 mmHg), normal (120–129 mmHg), high normal (130–139 mmHg), and abnormal (≥140 mmHg), according to ESC classification (Williams et al., 2018).

**TABLE 2:** Characteristics of population by systolic blood pressure levels (classified in four groups)

Parameters	Systolic blood pressure (mmHg)				*p*
	<120 *(optimal)*	120–129 *(normal)*	130–139 *(high normal)*	≥140 *(abnormal)*	
Overall population					
Number of subjects (*n* = 551)	*n* = 417 *(75.7%)*	*n* = 86 *(15.6%)*	*n* = 37 (*6.7%)*	*n* = 11 *(2%)*	*p*
SBP (mmHg)	106 ± 7	122 ± 3	132 ± 2	151 ± 15	—
Age (years)	22.1 ± 2.5	22.4 ± 2.4	23.7 ± 2.6	23.1 ± 3.3	.002
BMI (kg/m^2^)	21.4 ± 2.5	21.2 ± 2.3	21.3 ± 2.4	24.6 ± 5.3	.06
HR (bpm)	65 ± 11	66 ± 11	70 ± 13	69 ± 9	.07
Women					
Number of subjects (*n* = 160)	*n* = 144 *(90%)*	*n* = 11 *(6.9%)*	*n* = 4 *(2.5%)*	*n* = 1 *(0.6%)*	*p*
SBP (mmHg)	102 ± 7	123 ± 3	130 ± 0	150 ± 0	—
Age (years)	21.2 ± 2.1	20.1 ± 0.9	22.8 ± 2.9	23 ± 0	.13
BMI (kg/m^2^)	21.6 ± 3.3	22.6 ± 4.3	23.5 ± 3.8	39 ± 0	.23
HR (bpm)	71 ± 12	74 ± 16	83 ± 17	66 ± 0	.41
Men					
Number of subjects (*n* = 391)	*n* = 273 *(69.8%)*	*n* = 75 *(19.2%)*	*n* = 33 *(8.4%)*	*n* = 10 *(2.6%)*	*p*
SBP (mmHg)	108 ± 6	122 ± 3	132 ± 3	151 ± 16	—
Age (years)	22.6 ± 2.5	22.7 ± 2.3	23.8 ± 2.6	23.1 ± 3.5	.07
BMI (kg/m^2^)	21.2 ± 2	21 ± 1.9	21 ± 2.1	23.2 ± 2.5	.08
HR (bpm)	62 ± 10	65 ± 9	68 ± 12	70 ± 9	.0004

Note

Data expressed as means ± standard deviation.

Abbreviations: <, inferior; ≥, superior or equal; BMI, body mass index; DBP, diastolic blood pressure; HR, heart rate; SBP, systolic blood pressure.

In the overall population, the age distribution is found significantly different between SBP groups (Table 2). Because of the fewer sample size observed in some SBP groups (Table 2), SBP is considered in two groups (Table 3): normal group that includes people with SBP < 130 mmHg (including optimal and normal groups) and abnormal group with SBP ≥ 130 mmHg (including high normal and abnormal groups).

**TABLE 3:** Overall population characteristics by systolic blood pressure levels (classified in two groups)

Parameters	Systolic blood pressure (mmHg)	
	<130	≥130
Number of subjects (*n* = 551)	*n* = 503 *(91.3%)*	*n* = 48 *(8.7%)*
SBP (mmHg)	109 ± 9	136 ± 11
Age (years)	22.2 ± 2.4	23.6 ± 2.7
BMI (kg/m^2^)	21.3 ± 2.5	22.1 ± 3.5
HR (bpm)	65 ± 11	70 ± 12

Note

Data expressed as means ± standard deviation.

Abbreviations: <, inferior; ≥, superior or equal; BMI, body mass index; HR, heart rate; SBP, systolic blood pressure.

After controlling gender and BMI, the increase in the age of 1 year significantly increases by 20% the risk of having an SBP ≥ 130 mmHg (OR = 1.20, *p* = .002). After adjusting for gender and age, the increase in the BMI of 1 kg/m^2^ is significantly correlated with the risk of having an SBP ≥ 130 mmHg, (OR = 1.17, *p* = .008). Compare to women, men have 3.8 times higher risk of having an SBP ≥ 130 mmHg, after stratifying by age and BMI (OR = 3.8, *p* = .01).

Among subjects with SBP <120 mmHg (table 2), men have higher age (*p* < .0001) and SBP (*p* < .0001) compare to women. When controlling variables’ effects in linear regression, we noticed that only gender and BMI have a significant effect on SBP variation in this group. Indeed, with similar age and BMI, men have a 5.22 mmHg higher SBP than women (coef = 5.22, *p* < .001). After adjusting for age and gender, each 1 kg/m^2^ increase in BMI leads to 0.42 mmHg increase in SBP (coef = 0.42, *p* = .001).

### 3.3 Diastolic blood pressure (DBP)

In Table 4, DBP is presented in four (4) groups, optimal (<80 mmHg), normal (80–84 mmHg), high normal (85–89 mmHg), and abnormal (≥90 mmHg), according to ESC classification (Williams et al., 2018).

**TABLE 4:** Population characteristics by diastolic blood pressure levels (classified in four groups)

Parameters	Diastolic blood pressure (mmHg)				*p*
	<80 *(optimal)*	80–84 *(normal)*	85–89 *(high normal)*	≥90 *(abnormal)*	
Overall population					
Number of subjects (*n* = 551)	*n* = 462 *(83.8%)*	*n* = 65 *(11.8%)*	*n* = 8 *(1.5%)*	*n* = 16 *(2.9%)*	*p*
DBP (mmHg)	68 ± 5	81 ± 1	87 ± 2	94 ± 9	—
Age (years)	22.2 ± 2.5	22.3 ± 2.2	23.3 ± 3	25.1 ± 3.1	.002
BMI (kg/m^2^)	21.4 ± 2.4	21.7 ± 3.5	22.6 ± 2.9	20.8 ± 2.3	.42
HR (bpm)	65 ± 11	68 ± 12	74 ± 9	66 ± 10	.05
Women					
Number of subjects (*n* = 160)	*n* = 140 *(87.5%)*	*n* = 16 *(10%)*	*n* = 1 *(0.6%)*	*n* = 3 *(1.9%)*	*p*
DBP (mmHg)	68 ± 5	80 ± 1	86 ± 0	92 ± 3	—
Age (years)	21.2 ± 2.1	21.1 ± 1.4	20 ± 0	23 ± 6.1	.92
BMI (kg/m^2^)	21.7 ± 3.3	23.8 ± 5.6	22.3 ± 0	18.6 ± 0.8	.08
HR (bpm)	72 ± 12	66 ± 12	80 ± 0	72 ± 8	.07
Men					
Number of subjects (*n* = 391)	*n* = 322 *(82.4%)*	*n* = 49 *(12.5%)*	*n* = 7 *(1.8%)*	*n* = 13 *(3.3%)*	*p*
DBP (mmHg)	68 ± 6	81 ± 1	87 ± 2	95 ± 10	—
Age (years)	22.6 ± 2.5	22.8 ± 2.3	23.7 ± 2.9	25.5 ± 2.1	.0007
BMI (kg/m^2^)	21.2 ± 2	21 ± 2	22.6 ± 3.1	21.3 ± 2.3	.63
HR (bpm)	62 ± 10	68 ± 12	73 ± 9	64 ± 10	.0005

Note

Data expressed as means ± standard deviation

Abbreviations: <, inferior; ≥, superior or equal; BMI, body mass index; DBP, diastolic blood pressure; HR, heart rate; SBP, systolic blood pressure.

In the overall population, the age distribution is found significantly different between DBP groups (Table 4).

Because of the fewer sample size observed in some DBP groups (Table 4), DBP is considered in two groups (Table 5): normal group that includes people with DBP < 85 mmHg (including optimal and normal groups) and abnormal group with DBP ≥ 85 mmHg (including high normal and abnormal groups). After adjusting for gender and BMI, each rise of 1 year in the age is significantly associated with an increase of 36% in the risk of having a DBP ≥ 85 mmHg, (OR = 1.36, *p* = .0001). Gender and BMI have no significant influence on DBP variation.

**TABLE 5:** Overall population characteristics by diastolic blood pressure levels (classified in two groups)

Parameters	Diastolic blood pressure (mmHg)	
	<85	≥85
Number of subjects (*n* = 551)	*n* = 527 *(95.6%)*	*n* = 24 *(4.4%)*
DBP (mmHg)	69 ± 7	92 ± 8
Age (years)	22.2 ± 2.4	24.5 ± 3.1
BMI (kg/m^2^)	21.4 ± 2.6	21.4 ± 2.6
HR (bpm)	66 ± 12	69 ± 10

Note

Data expressed as means ± standard deviation

Abbreviations: <, inferior; ≥, superior or equal; BMI, body mass index; DBP, diastolic blood pressure; HR, heart rate.

In men population, age distribution is found significantly different between DBP groups (Table 4). After controlling BMI, the increase in the age of 1 year is significantly correlated with an increase of 41% in the risk of having a DBP ≥ 85 mmHg (OR = 1.41, *p* = .0001). BMI has no significant influence on DBP variations.

In women population, no difference is observed between DBP groups (Table 4).

### 3.4 Blood pressure and family history

Results show that in the overall population, subjects with a family history of abnormal high blood pressure have a significantly higher DBP than those with no abnormal high blood pressure in family history. This significant difference is also found in men population but not in women population (Table 6).

**TABLE 6:** Study Population characteristics by Family History

Parameters	Family history of abnormal high blood pressure		*p*
	YES	NO	
Overall population			
Number of subjects (*n* = 551)	*n* = 177 *(32.1%)*	*n* = 374 *(67.9%)*	*p*
Age (years)	22.1 ± 2.6	22.4 ± 2.5	.26
SBP (mmHg)	111 ± 12	111 ± 12	.68
DBP (mmHg)	72 ± 7	70 ± 8	.01
Women			
Number of subjects (*n* = 160)	*n* = 65 *(40.6%)*	*n* = 95 *(59.4%)*	*p*
Age (years)	21.1 ± 1.9	21.4 ± 2.2	.26
SBP (mmHg)	105 ± 9	105 ± 11	.88
DBP (mmHg)	71 ± 7	70 ± 7	.34
Men			
Number of subjects (*n* = 391)	*n* = 112 *(28.6%)*	*n* = 279 *(71.4%)*	*p*
Age (years)	22.7 ± 2.7	22.7 ± 2.4	.94
SBP (mmHg)	115 ± 12	113 ± 12	.10
DBP (mmHg)	72 ± 8	70 ± 9	.01

Note

Data expressed as means ± standard deviation.

Abbreviations: BMI, body mass index; DBP, diastolic blood pressure; SBP, systolic blood pressure.

## 4 DISCUSSION

Therefore, the purpose of this study was to determine the blood pressure profile according to age and anthropometric parameters in healthy young black African students aged between 18 and 30 years, living in Côte d’Ivoire, West Africa.

### 4.1 Study limitations

Taking into account the genetic and environmental aspects of populations, our results can not be extrapolated to black Africans in general. Further studies are needed to compare black Africans of several countries.

The classification of SBP and DBP, each into four groups, significantly reduces the number of subjects in a certain group without possible statistical testing.

### 4.2 Systolic blood pressure

In the overall population, higher SBP (≥130 mmHg) was positively associated with age, BMI, and male gender. In SBP range <120 mmHg, SBP was significantly correlated with BMI and male gender.

Systolic blood pressure (SBP) is determined by cardiac output, vascular resistance of large arterial trunks, and peripheral reflection waves (Asmar, 2007). Over the years, arteries undergo structural physiological and functional changes that induce arterial stiffness (Franklin et al., 1997; Grossen, 2002; Lakata, 1986).The result is vasoconstriction with an increase in peripheral vascular resistance (Grossen, 2002). These various modifications of artery wall could explain the increase in SBP with age in our overall population aged between 18 and 30 years old. In black American subjects, SBP increases with age between 20 and 35, in contrast to the caucasian subjects in whom SBP remains stable until the age of 35 years (Kishi et al., 2015; NCD, 2017). This difference observed between black and caucasian subjects could be explained by an early increase of arterial stiffness in the black subject (Morris et al., 2013; Su et al., 2014).

Concerning the correlation of SBP with BMI, in line with our results, among 2,726 cameroonian aged 18 years or above, Choukem and al (Choukem et al., 2017) showed that each 1 kg/m^2^ increase in BMI was associated with an 11% increase in the prevalence of abnormal high blood pressure (*p* = .013). Also, Yano et al. (2017), in a study enrolling 2,473 black adults between 18 and 30 years old, found that higher SBP was significantly associated with higher BMI (coef = 0.22, *p* < .001). For Hosseini et al. (2015), in healthy individuals, blood pressure (BP) changes with body mass. The relationship between BMI and blood pressure has been shown to continue down to at least 18 kg/m^2^ (Whitlock et al., 2009). Adipose tissue has been traditionally considered a fat‐storage organ but is now known to have an active role in systemic metabolism through the active secretion of adipokines or obesity hormones (Gustafson, Hammarstedt, Andersson, & Smith, 2007; Hiuge‐Shimizu et al., 2012). Mechanisms behind the association of adiposity with blood pressure are complex and include the dysfunctional adipose tissue‐promoting activation of the sympathetic nervous system and the renin‐angiotensin‐aldosterone pathway, and adiponectin deficiency reducing nitric oxide production and increasing systemic inflammation and oxidative stress (Dorresteijn, Visseren, & Spiering, 2012; Kotsis, Stabouli, Papakatsika, Rizos, & Parati, 2010). Several studies are needed to explain the role of these systems by population.

Consistent with our results on SBP and gender, Yano and al, in a mixed population of 2,473 black Americans aged between 18 and 30 years, found a higher SBP in men than in women, respectively, 115.5 ± 11 mmHg and 108 ± 10 mmHg (*p* < .001). Similarly, Choukem and al (Choukem et al., 2017), in a mixed population of 2,726 young Cameroonian aged 21.8 ± 2.4 years, noticed that men had a higher SBP than women (120 ± 12 mmHg vs. 113 ± 12 mmHg). Young women typically have lower blood pressure than men (Joyner, GunnarWallin, & Charkoudian, 2016). Arterial blood pressure is a key regulated variable in the cardiovascular system. Its determinants can vary by gender. In young men, there is a direct relationship between muscle sympathetic nerve activity (MSNA) and total peripheral resistance: the two main factors determining arterial pressure balanced each other to maintain normal blood pressure. However, in young women there is no relationship between MSNA and total peripheral resistance (or cardiac output) because β‐adrenergic vasodilator mechanisms offset α‐adrenergic vasoconstriction (Joyner et al., 2016). Moreover, women and men differ in their left ventricular dimensions and functions. Women have smaller left ventricle and left ventricular systolic ejection volume than men, a higher heart rate and greater left ventricular elastance (systolic and diastolic), and arterial elastance, a stiffer aorta, and earlier wave reflections (Beale, Meyer, Marwick, Lam, & Kaye, 2018; Redfield, Jacobsen, Borlaug, Rodeheffer, & Kass, 2005). A study on the influence of these parameters by gender and SBP level in our black African population would help us to better understand SBP values.

### 4.3 Diastolic blood pressure

In our overall population, DBP was optimal (<80 mmHg) and was similar between men and women. DBP was positively correlated with age. This DBP/age relationship has been observed in men population. Moreover, men had a significant increase of DBP when having an abnormal high blood pressure family history. However, in women, this DBP/age relationship was not observed. In line with our results, Choukem and al (Choukem et al., 2017) found optimal and similar DBP between men and women (70 ± 10 mmHg) among 2,726 cameroonian aged 18 years or above. For Yano and al (Yano et al., 2017), in black Americans aged between 18 and 30 years, DBP was correlated with age (coef = 0.19, *p* < .001) and was significantly higher in men than in women (70.6 ± 10.4 mmHg; 67.5 ± 9.4 mmHg; *p* < .001). DBP is dependent on peripheral arterial resistances, duration of diastole, and little on the wall condition of large arterial trunks (Asmar, 2007). Structural and functional changes in arteriolar parietal walls with age lead to a decrease in the arteriolar radius with a consequent reduction in these vessels diameter (Grossen, 2002). This could explain the increase of DBP with age. Vascular resistance responses and regulation involve endothelium‐derived relaxing and constricting factors are influenced by gender (Ait‐Oufella, Maury, Guidet, & Offenstadt, 2008). Indeed, in women, estrogens help the release of vasodilator nitric oxide in counter to androgens in men (Stanhewicz, Wenner, & Stachenfeld, 2018). Moreover, vasoconstrictor endothelin‐1 endothelial production is higher in men (Polderman et al., 1993). Further studies on DBP levels and endothelial factors production correlation in different populations could help to better understand DBP variations according to gender.

In our study, DBP was not associated with BMI. For Whitlock and al, (Whitlock et al., 2009) BMI, the most commonly used marker of adiposity, is strongly related to blood pressure. Also, for Yano et al. (2017), in their study enrolling 2,473 black adults 18 to 30 years old, higher DBP was significantly associated with higher BMI (coef = 0.18, *p* < .001). The relevance of adiposity to blood pressure varies between populations (Gnatiuc et al., 2017). Moreover, different markers of adiposity also differ in their relevance to blood pressure (Gnatiuc et al., 2017). It would be interesting to study them in our black African population to seek a possible relationship between adiposity and DBP.

## 5 CONCLUSION

In this population of young black African students of Côte d’Ivoire aged between 18 and 30 years, systolic blood pressure is positively associated with male gender, age, and body mass index. Whereas diastolic blood pressure is correlated only with age. These results strengthen the need to develop preventive measures against high blood pressure in Côte d’Ivoire from the age of 18.

## CONFLICT OF INTEREST

The authors have no conflicts of interest.

## AUTHOR'S CONTRIBUTION

**Edwige Siransy‐Balayssac:** Conception of the work, Analysis, and interpretation of data for the work, Drafting of the work, Approved the final version of the manuscript Agree to be accountable for all aspects of the work; **Soualiho Ouattara:** Analysis, and interpretation of data for the work, Drafting of the work, Approved the final version of the manuscript, Agree to be accountable for all aspects of the work; **Téniloh Augustin Yéo:** Conception of the work, Acquisition, and analysis of data for the work, Drafting of the work, Approved the final version of the manuscript, Agree to be accountable for all aspects of the work; **Aya Liliane Kondo:** Acquisition of data for the work, Drafting of the work, Approved the final version of the manuscript, Agree to be accountable for all aspects of the work; **Massiré Touré:** Acquisition, analysis, and interpretation of data for the work, Drafting of the work, Approved the final version of the manuscript, Agree to be accountable for all aspects of the work; **Cyrille Serges Dah:** Conception of the work, Revising it critically for important intellectual content, Drafting of the work, Approved the final version of the manuscript, Agree to be accountable for all aspects of the work.
